# The mediating role of resilience in the relationship between social support and quality of life among patients after radical cystectomy: A structural equation model analysis

**DOI:** 10.1002/nop2.1408

**Published:** 2022-11-03

**Authors:** Shuhui Yu, Xiuyu Yao, Yonghui Sang, Lingling Yu, Yiru Shen, Xinyan Che, Yanming Ding, Yanbo Huang

**Affiliations:** ^1^ Department of Urology Peking University First Hospital Beijing China; ^2^ Peking University Health Science Centre for Evidence‐Based Nursing: A Joanna Briggs Institute Affiliated Group Beijing China; ^3^ School of Nursing Peking Union Medical College Beijing China; ^4^ Nursing Department Peking University First Hospital Beijing China

**Keywords:** cystectomy, quality of life, resilience, social support, structural equation model, urostomy patients

## Abstract

**Aim:**

This study aimed to examine the relationship between social support and quality of life in urostomy patients and identify the mediating role of resilience in that relationship.

**Design:**

A cross‐sectional design.

**Methods:**

Participants included 232 patients who were recruited from a tertiary hospital in Beijing during March 2020 and August 2020. They completed questionnaires about perceived social support, resilience and ostomy‐related quality of life. Structural equation modelling was performed to analyse the data.

**Results:**

The mean age of patients was 65.79 (*SD =* 8.67) years, and the mean length of time after surgery was 42.14 (*SD =* 15.76) months. Urostomy patients' quality of life, social support and resilience were all above moderate. Social support had a positive direct effect on the quality of life and a positive indirect effect on the quality of life through the mediating role of resilience.

## 1 INTRODUCTION

Bladder cancer has become the most common cancer of the urinary system. In 2015, the prevalence of bladder cancer in China reached 2.88%, and it was ranked the seventh most common form of cancer among men (Zheng et al., 2019). In 2020, in the United States, the estimated number of deaths from bladder cancer was 33,820 (Rebecca et al., 2020). Radical cystectomy remains the standard treatment for muscle‐invasive bladder cancer, and the use of an ileal conduit is the preferred choice of many patients who undergo uncontrolled urinary diversion (Sean et al., 2002). Studies have shown that the diagnosis and surgical treatment of bladder cancer can change patients' daily activities (Tyson & Barocas, 2018; Yang et al., 2016). For example, after surgery, patients have to carry an ostomy bag, which has been found to provoke social isolation and many negative emotions, such as stress, anxiety, fear and depression (Ayaz‐Alkaya, 2019). These physical and emotional responses can significantly impair the quality of life (QOL) of patients (Zhang et al., 2019). The assessment of the QOL in ostomy patients was recommended in the latest practice guidelines issued by the Registered Nurses' Association of Ontario, Canada (Registered Nurses' Association of Ontario, 2019).

Resilience refers to an individual's adaptability in the face of significant change, adversity or risk. Resilient individuals can ‘bounce back’ from stressful events quickly and effectively (Stainton et al., 2019). Individual resilience has been shown to be an important factor influencing adult cancer patients' response to adversity (Luo et al., 2020). Studies have shown a positive correlation between QOL and resilience; for example, resilience was associated with improved QOL in patients with breast cancer and patients who had undergone a drainage enterostomy (Ristevska‐Dimitrovska et al., 2015; Temprado et al., 2020). Moreover, patients with similar disease and status have been found to have different levels of QOL, which may be due to their varying levels of resilience (Harms et al., 2019). An increasing body of research has suggested the mediating role of resilience in health‐related outcomes. For example, Wu et al. (2018) investigated the mediating role of resilience in the relationship between social support and health‐related QOL among Chinese rural elders in nursing homes, and Dong et al. (2017) found a mediating role of resilience in the relationship between social support and posttraumatic growth among colorectal cancer survivors with permanent intestinal ostomies.

Social support can be defined in many ways. According to theories that stress social networks, social support refers to the care and support that are accessible to an individual through ties to other individuals, groups and the larger community (Gottlieb & Bergen, 2010). A literature review indicated that social support systems are important protective factors for individuals experiencing adversity or stressful events (Wang et al., 2014). There are two widely accepted frameworks about the role of social support in health‐related outcomes (Cohen & Wills, 1985): the main effects model, which states that social support has a direct and positive effect on health‐related outcomes (Wang et al., 2014), and the buffering model, which states that social support often works through a person's internal cognition (Aneshensel & Stone, 1982). Recent research findings have found that social support is associated with psychological stress and that resilience may mediate between the two.

Patients with rectal cancer who have an ostomy for 1–10 years after surgery tend to report significantly lower physical functioning, social functioning and QOL, compared with patients without an ostomy (Mols et al., 2014). The risk of complications from stoma formation is lifelong, but the incidence of complications is highest in the first 5 years after surgery (Londono‐Schimmer et al., 1994). For example, parastomal hernia, the most common complication of ostomy patients, has an incidence rate exceeding 30% in the first year after surgery and can greatly affect patients' QOL (Antoniou et al., 2018). Thus, medical service providers should take note of ostomy patients' QOL, especially in the first few years after surgery. To the best of our knowledge, only a few studies have examined the relationship among social support, resilience and QOL among patients with urinary ostomies (Wu et al., 2018). Nurses may benefit from learning new ways of improving patients' health outcomes and their QOL. Therefore, this study sought to explore the mediating role of resilience in the relationship between social support and QOL among ostomy patients more than 1 year after surgery.

### 1.1 Research questions

The present study aimed to answer three main questions: (a) What are the levels of resilience, social support and QOL among outpatients with urostomy? (b) What are the relationships between these three variables? (c) Does resilience mediate the relationship between social support and QOL?

## 2 THE STUDY

### 2.1 Research design

A cross‐sectional survey design was used in this study.

### 2.2 Patients

The participants comprised patients who were diagnosed with bladder cancer in one hospital in Beijing. The inclusion criteria were (a) persons aged 18 years and older with urostomy, (b) patients with single bladder cancer who underwent radical cystectomy and had an ileal conduit and (c) patients who had ostomy pouches for more than 12 months. Patients were excluded if they had more than one tumour or another benign disease, such as interstitial cystitis or pelvic lipomatosis, or if they were in the hospital for a urostomy. A total of 415 patients were initially enrolled in the study; 129 patients did not complete the follow‐up and 58 patients died; therefore, data from 232 patients were analysed. The response rate for this study was 68.91%.

### 2.3 Procedure

We used a convenience sampling technique to recruit ostomy patients who had undergone an ileal conduit between January 2014 and December 2018, all of whom had regular follow‐up appointments for 6 months in the ostomy outpatient department. The ostomy nurse practitioner provided information packets to the patients at the end of the follow‐up, which included information about ostomy complications, how to deal with those complications, potential outcomes and the pouching system. This study was conducted between March 2020 and August 2020 at a tertiary hospital in Beijing. The participants spent 25 min completing a questionnaire in the outpatient department. A designated person provided support for the patients who had any queries.

### 2.4 Measures

We mainly used suitable scales to assess patients for their levels of resilience, social support and quality of life.

The Chinese version of the 10‐item Connor–Davidson Resilience Scale (CD‐RISC) (Wang et al., 2010) was used to assess participants' resilience. The scale consists of 10 items rated on a 5‐point Likert‐type scale (0 = not true at all to 4 = true nearly all the time), with total scores ranging from 0–40. Higher scores indicated higher resilience. Wang et al. (2010) showed that this scale had good internal consistency (Cronbach's alpha = 0.91) and test–retest reliability (*r* = .90). The Cronbach's alpha of this study was 0.772.

The Chinese version of the Perceived Social Support Scale (PSSS), a 12‐item measure developed by Zimet et al. (1990) and translated by Wang et al. (2010), was used to determine participants' perceived social support. The PSSS includes three subscales: family support (4 items), friend support (4 items) and other support (4 items). Responses are rated on a 7‐point Likert‐type scale (1 = strongly disagree to 7 = strongly agree). Scores range from 12–84, with a score of 12–35 indicating low perceived support, 36–61 indicating moderate perceived support and 62–84 indicating high perceived support. Cronbach's alpha was 0.931. The Cronbach's alpha of this study was 0.934.

The Chinese version of the City of Hope Quality of Life Ostomy Questionnaire (C‐COH), which was translated by Gao et al. (2013) from the original developed by Grant et al. (2004), was used to assess participants' QOL. The C‐COH includes 32 items across four domains: physical well‐being (7 items), psychological well‐being (11 items), social well‐being (9 items) and spiritual well‐being (5 items). Each item is assigned values ranging from 0–10. The total QOL score is obtained by adding all items and dividing them by 32. Cronbach's alphas were 0.95, 0.860, 0.885, 0.864 and 0.686 for the total score, physical well‐being, psychological well‐being, social well‐being and spiritual well‐being respectively. The Cronbach's alpha of this study was 0.880.

### 2.5 Data analysis

Data analyses were performed using SPSS version 20.0 and AMOS version 22.0. All data were checked and analysed by two researchers. Descriptive statistics were used to present the characteristics of the participants that were obtained from social‐ and disease‐related information and the score from the three questionnaires. Pearson's correlation coefficients were calculated to test the correlation among resilience, social support and QOL. Structural equation modelling was performed with AMOS to test the structural relationships among the variables (Byrne, 2016).

A hypothesized mediation model was specified with social support as the exogenous variable, resilience as the mediator and QOL as the endogenous variable. The model fit was assessed by examining the *χ* ^2^ statistic, with a *p*‐value greater than .05 indicating failure to reject the null hypothesis, the comparative fit index (CFI), the goodness of fit index (GFI), the root mean square error of approximation (RMSEA) and the *χ* ^2^/*df* ratio (Byrne, 2016). To analyse mediating effects and test their significance, we used the method developed by Wen (Wen & Ye, 2014). A *p‐*value of less than .05 was considered statistically significant.

### 2.6 Ethical considerations

This study was approved by the Ethics Committee of Peking University First Hospital in which the study was conducted (Number: 2015–977). The participants were told that they could withdraw from the study at any time and that the data would only be used for academic research. All participants provided written informed consent.

## 3 RESULTS

### 3.1 Characteristics of participants

Participants' characteristics are presented in Table 1. The mean age was 65.79 years (*SD* = 8.67, range = 21–50). The mean postoperative time of patients undergoing ostomy was 42.14 months (*SD* = 15.76). The shortest postoperative length was 18 months and the longest was 76 months. The mean body weight was 70.69 kg (*SD* = 11.28), and most of the participants were male (189; 81.47%). More than half of the participants relied on others for self‐care (118; 50.86%).

**TABLE 1:** Demographic and clinical characteristics (*N* = 232)

Item	Category	Frequency	Percent
Gender	Male	189	81.47
	Female	43	18.53
BMI classification	<19	9	3.88
	19–24	101	43.53
	24–29	100	43.10
	>29	22	9.48
Marital status	Married	230	99.14
	Single	2	0.86
Occupation	On‐the‐job	10	4.31
	Retired	118	50.86
	Peasantry	16	6.90
	Unemployed	28	12.07
	Others	60	25.86
Payment method of medical expenses	Public expense	21	9.05
	Medicare	190	81.90
	Self‐paying	21	9.05
Residence	Urban	191	82.33
	Countryside	41	17.67
Educational background	Elementary school and below	28	12.07
	Middle school	64	27.59
	High school	48	20.69
	Technical secondary school	23	9.91
	Bachelor	33	14.22
	Master	36	15.52
Living with	Spouse	214	92.24
	Children	18	7.76
Self‐care	Completely dependent on others	103	44.40
	Mostly dependent on others	15	6.47
	Mostly self‐care	4	1.72
	Completely self‐care	110	47.41
Stoma complication	N/A	169	72.84
	I/A	63	27.16
Expense for stoma per month (RMB)	<500	77	33.19
	500–1,000	112	48.28
	1,000–1,500	27	11.64
	1,500–2,000	16	6.90
Tumour recurrence	I/A	12	5.17
	N/A	220	94.83

### 3.2 Descriptive statistics of resilience, social support and quality of life

Table 2 presents the descriptive statistics for the three main variables. The scores on the three measurements were all above the average score for each scale, which indicated the stratified status of the participants. Regarding social support, family support (mean = 25.30, *SD* = 2.30) scored higher than the other two types of support, suggesting that family support was the main source of support. For QOL, physical health was rated the highest (mean = 8.68, *SD* = 1.13), whereas mental health was the lowest (mean = 7.80, *SD* = 1.00).

**TABLE 2:** The scores on three scales of social support, quality of life and resilience (*N* = 232)

Dimension	Items	Aggregate score	Average score ± *SD*
Social support	12	84	71.81 ± 8.02
Family support	4	28	25.30 ± 2.30
Friends support	4	28	23.19 ± 3.30
Others support	4	28	23.32 ± 3.34
Quality of quality	32	320	8.24 ± 0.91
Psychological well‐being	11	110	8.68 ± 1.13
Social well‐being	9	90	7.80 ± 1.00
Physical well‐being	7	70	8.37 ± 1.48
Spiritual well‐being	5	50	7.89 ± 1.11
Resilience	10	40	31.27 ± 5.27

### 3.3 Correlations among resilience, social support and quality of life

Correlations among resilience, QOL and social support are presented in Table 3. Resilience was positively correlated with QOL and social support in all three dimensions.

**TABLE 3:** Correlation analysis of resilience between social support and quality of life

	Resilience
Social support	0.490^***^
Friends support	0.511^***^
Others support	0.460^***^
Family support	0.306^***^
Quality of life	0.311^***^
Spiritual well‐being	0.231^***^
Social well‐being	0.187^**^
Psychological well‐being	0.272^***^
Physical well‐being	0.274^***^

**p < .05, ***p < .01.

### 3.4 Fit indices for the hypothesized model of resilience, social support and quality of life

A well‐fitting model was considered if the CFI and GFI values were both greater than 0.90 and the RMSEA was less than 0.10 (Michael & Robert, 1992). Additional goodness of fit was considered if the *χ* ^2^/*df* ratio was not greater than 5. The results indicated that the model fit the data well: *χ* ^2^ = 44.334, *p* = .001, CFI = 0.968, GFI = 0.957, RMSEA = 0.088 and *χ* ^2^/*df* = 2.771.

**FIGURE 1:**
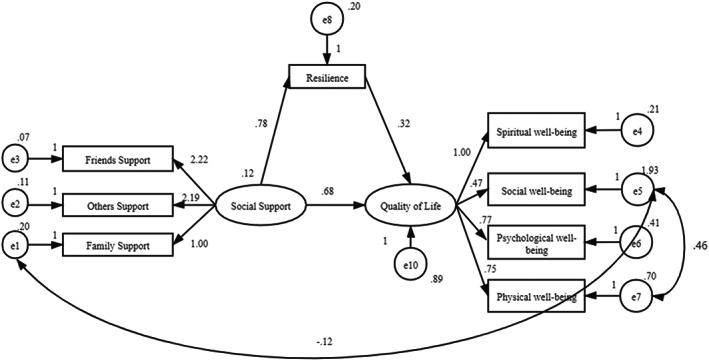
Analysis of the mediating effect of resilience on social support and quality of life

The results indicated that resilience was positively related to social support (*β* = 0.78, *p* < .01) and QOL (*β* = 0.32, *p* < .01), as shown in Figure 1. The total, direct and indirect effects of social support on coping are summarized in Table 4. The indirect effect of social support on QOL was 0.254 (*p* < .001), indicating that the mediating effect was statistically significant. Resilience played a partial mediating role (mediation effect rate of 0.254) in the relationship between social support and QOL.

**TABLE 4:** Mediating effect analysis of resilience on social support and quality of life

Effect	Variable relation	Point estimation	*SE*	Z	*p*
Total effect	Social support–quality of life	0.931	0.237	3.928	<.001
Indirect effect	Social support–resilience	0.785	0.11	7.136	<.001
	Resilience–quality of life	0.324	0.153	2.118	<.05
Direct effect	Social support–quality of life	0.677	0.243	2.786	<.05

## 4 DISCUSSION

An ostomy requires patients to adapt both physically and psychosocially, and it can affect an individual's QOL. Many factors may impact ostomy patients' QOL, such as coping strategies, socio‐economic factors, resilience, symptoms and support systems, and a guideline about ostomy management developed by the Wound, Ostomy and Continence Nurses Society indicated that further studies are needed to explore the effects of these factors. Therefore, the current study tested the influence of internal factors (resilience) and external factors (social support) on the QOL of urostomy patients. Our findings confirmed that social support had a direct effect on QOL, and resilience played a mediating role in the relationship between social support and QOL.

Quality of life scores in this study were higher than those reported by Pazar et al. (2015), whose study included patients who had undergone a urostomy operation with a mean postoperative period of 9.83 (*SD* = 2.34) months. This may be because an ostomy tends to have a negative impact on QOL, but the impact usually decreases over time (Krouse et al., 2009). One study showed that ostomy patients had significant declines in physical and mental health related to QOL compared to patients without cancer (Winters et al., 2018), indicating the importance of improving the QOL among this group.

The mediation analysis suggested that social support had a strong, positive direct effect on QOL, and higher social support was correlated with a higher QOL. These findings are similar to those of Leyk et al. (2014), who found a significant relationship between the level of social support and QOL in persons with permanent colostomies for more than 1 year. The longer the patients lived with colostomies, the greater the effect of social support. The PSSS refers mainly to patients' perceptions of social support, which can promote patients' recognition of the disease, enhance their ability to cope with trauma and improve their mental health levels. One study pointed out that social support had a negative effect on stress perception in patients with colorectal cancer (Costa et al., 2017).

The level of perceived social support was above average in this study, and the main source of social support was familial. The prominence of family support may be explained in terms of filial piety and the older adult care policy in China, where family members usually assume the responsibility of caring for older patients. In addition, family members acquire the skills required to provide ostomy care to patients because patients and their family members learn about ostomy care from specialized nurses at the hospital. Shapiro (2018) pointed out that cancer survivorship started from the time of diagnosis and lasted through the lifespan, and should include family members, friends and caregivers because they provide physical and emotional support to the cancer survivor. In the future, the specialized nurse will strengthen to encourage patients to seek help from their families and consider other means of social support through the community, ostomy clinics and fellowships and network information exchanges (e.g. WeChat and other social media platforms). Improving their levels of social support and adapting to ostomy conditions helps patients to return to normal life.

The mediation analysis revealed a mediating role of resilience in the relationship between social support and QOL, indicating that resilience played a significant role to strengthen the positive influences of confrontation and perceived social support. This was in accordance with the findings of Zhou et al. (2022). Another study found that resilience had a moderately positive effect on the physical, social and emotional aspects of QOL and a negative effect on stress perception in patients with colorectal cancer (Costa et al., 2017). Kasser & Zia (2020) found that resilience was strongly correlated with optimism and self‐efficacy, showed strong inverse correlations with fatigue and pain and had a statistically significant mediation effect on QOL. Resilience gave patients with cancer a stronger fighting spirit and made it easier for them to accept and adjust to psychological damage (Daly, 2020). The level of resilience in the present study was moderate; thus, methods suitable for Chinese ostomy patients should be used to improve their resilience.

### 4.1 Limitations

This study has some limitations. First, since convenience sampling was used to recruit participants and the postoperative length of patients was more than 1 year, the results may not be generalizable to patients with postoperative lengths of less than 1 year. Second, this was a cross‐sectional study; therefore, causal inferences could not be made. Additionally, all patients were from a single‐centre hospital in Beijing, China, which limited the number of patients compared with tertiary hospitals. It is recommended that future research use a longitudinal design to enable a better understanding of social support, resilience and QOL. In addition, future studies should use an experimental design to identify strategies that are effective in promoting social support, resilience and QOL.

## 5 CONCLUSIONS

The QOL of urostomy patients after surgery has been receiving increasing attention. The results of this study showed that social support was positively related to QOL among urostomy patients for postoperative periods of more than 1 year. In addition, this study confirmed the mediating role of resilience in the relationship between social support and QOL. These findings revealed that social support and resilience worked together to affect QOL and provide a reference for the development of interventions to promote QOL among ostomy patients.

## 6 RELEVANCE TO CLINICAL PRACTICE

The results show that social support and resilience contribute to the QOL of older patients who have been fitted with urostomies for more than 1 year after surgery. These findings have potential implications for practice and theoretical research. First, social support was shown to be derived mainly from family, so specialized nurses and rehabilitation personnel should teach patients and their families about ostomy care in the hospital setting and encourage patients to seek support from friends and others through social networks and transitional care. Second, social support and resilience should be measured in the general management of urostomy patients to provide health providers with information about these indicators of overall QOL. Finally, resilience is a dynamic process. Although there are some studies on improving levels of resilience, such as mindfulness, there is no firm conclusion regarding the most effective intervention type. Thus, future studies should investigate this in more detail to determine the most effective intervention type.

## AUTHOR CONTRIBUTIONS

Study design – Shuhui Yu, Xiuyu Yao, Xinyan Che, Yanming Ding and Yanbo Huang. Data collection – Xinyan Che and Yonghui Sang. Data analysis – Lingling Yu and Yiru Shen. Manuscript writing – Shuhui Yu, Xiuyu Yao, Xinyan Che and Yanming Ding.

All those listed as authors should qualify for authorship by meeting all four of the following criteria:

1. Have made substantial contributions to conception and design, acquisition of data or analysis and interpretation of data;
2. Been involved in drafting the manuscript or revising it critically for important intellectual content;
3. Given final approval of the version to be published. Each author should have participated sufficiently in the work to take public responsibility for appropriate portions of the content and
4. Agreed to be accountable for all aspects of the work in ensuring that questions related to the accuracy or integrity of any part of the work are appropriately investigated and resolved.

## FUNDING INFORMATION

Peking University Evidence‐Based Nursing Research Fund (XZJJ‐2022‐02).

## CONFLICT OF INTEREST

None.

## Data Availability

The data that support the finding of this study are available on request from the corresponding author.
